# Supplementary material for: Octopus-Inspired Soft Robot for Slow Drug Release
Source: Biomimetics (Basel). 2024 Jun 4;9(6):340. doi: 10.3390/biomimetics9060340 (PMC11202092; doi:10.3390/biomimetics9060340)
Supplement: Supplementary file 1 [file biomimetics-09-00340-s001.zip › Supporting information.pdf]

biomimetics MDPI

Supplementary Materials for

Octopus-inspired Soft Robot for Slow Drug Release

Dingwen Tong *et al.*

Correspondence:
xinjianfan@suda.edu.cn
zhanyang@suda.edu.cn

The PDF file includes:

Figures S1 to S5

Other Supplementary Material for this manuscript includes the following:

Videos S1 to S9

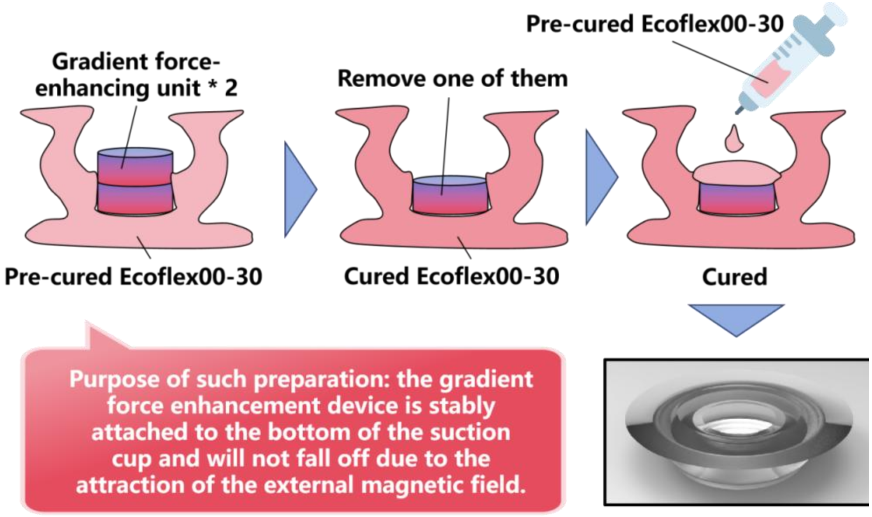

Figure S1. Gradient force-enhancing unit Assembly Procedure

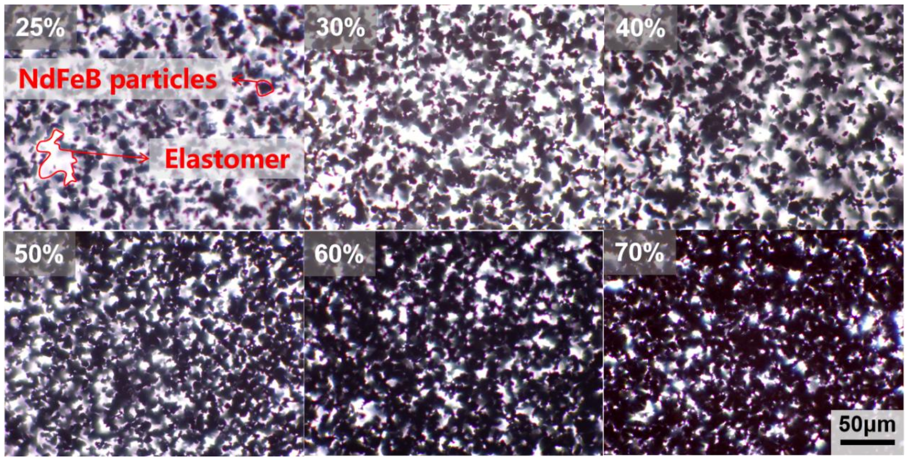

Figure S2. Optical micrographs of NdFeB particles mixed with Ecoflex00-50 at different mass fractions.

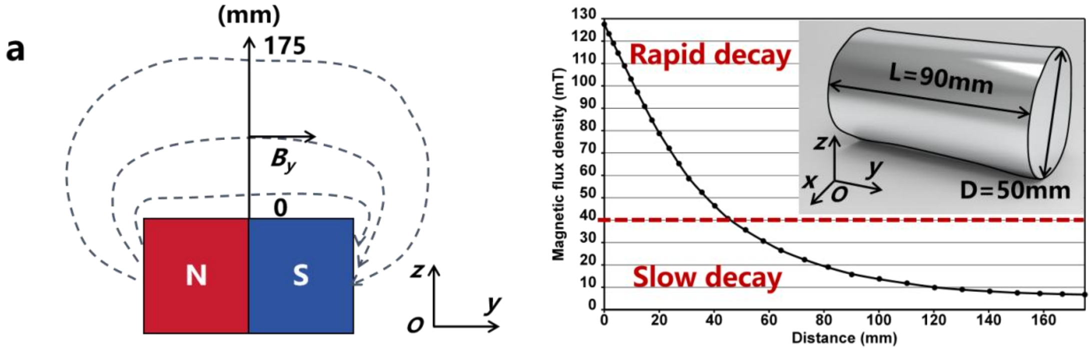

Figure S3. Cont.

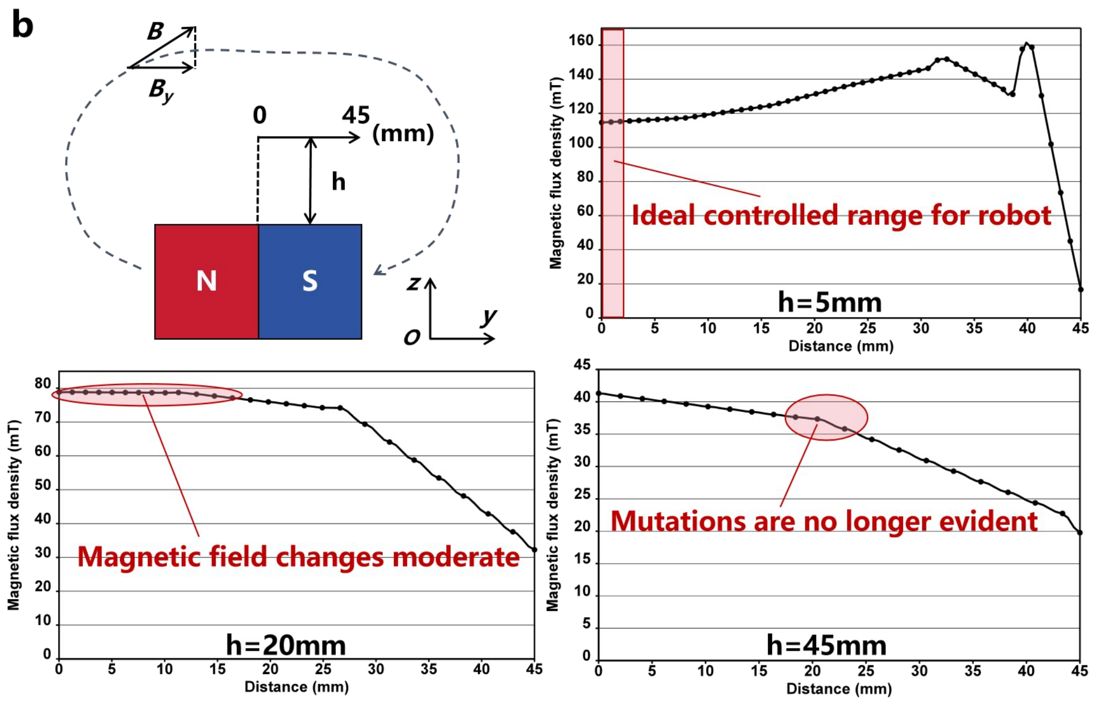

Figure S3. Spatial distribution of the simulated magnetic flux intensity for the quick scrolling driver (COMSOL Multiphysics 6.0, COMSOL, Sweden). (a) By distribution in z-direction. (b) By distribution in y-direction for different height.

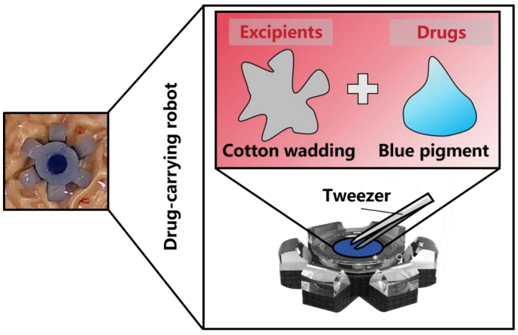

Figure S4. Simulation of drugs stored inside the sucker by cotton wadding and blue pigment.

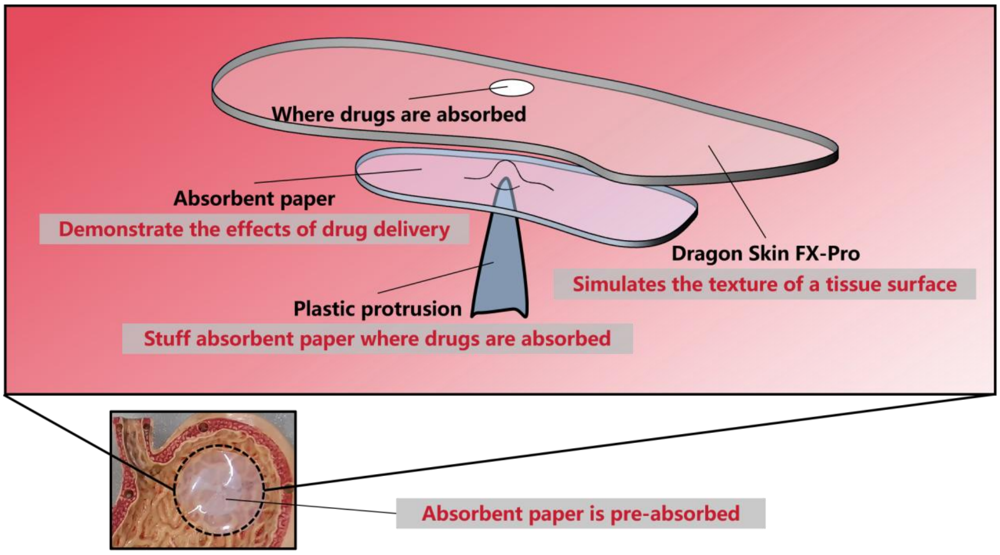

Figure S5. Preparation of the drug delivery target site.
